# Why Mental Illness Diagnoses Are Wrong: A Pilot Study on the Perspectives of the Public

**DOI:** 10.3389/fpsyt.2022.860487

**Published:** 2022-04-29

**Authors:** Yi-Sheng Chao, Chao-Jung Wu, Yi-Chun Lai, Hui-Ting Hsu, Yen-Po Cheng, Hsing-Chien Wu, Shih-Yu Huang, Wei-Chih Chen

**Affiliations:** ^1^Independent Researcher, Montreal, QC, Canada; ^2^Département d’Informatique, Université du Québec à Montréal, Montréal, QC, Canada; ^3^National Yang Ming Chiao Tung University Hospital, Yilan, Taiwan; ^4^Changhua Christian Hospital, Changhua, Taiwan; ^5^National Taiwan University Hospital, New Taipei City, Taiwan; ^6^Department of Anesthesiology, Shuang Ho Hospital, Taipei Medical University, New Taipei City, Taiwan; ^7^Department of Anesthesiology, School of Medicine, College of Medicine, Taipei Medical University, Taipei, Taiwan; ^8^Department of Chest Medicine, Taipei Veterans General Hospital, Taipei, Taiwan; ^9^Institute of Emergency and Critical Care Medicine, National Yang Ming Chiao Tung University, Taipei, Taiwan

**Keywords:** mental illness, Diagnostic and Statistical Manual of Mental Disorders (DSM), International Classification of Diseases (ICD), assumption, confidence

## Abstract

**Background:**

Mental illness diagnostic criteria are made based on assumptions. This pilot study aims to assess the public’s perspectives on mental illness diagnoses and these assumptions.

**Methods:**

An anonymous survey with 30 questions was made available online in 2021. Participants were recruited via social media, and no personal information was collected. Ten questions focused on participants’ perceptions regarding mental illness diagnoses, and 20 questions related to the assumptions of mental illness diagnoses. The participants’ perspectives on these assumptions held by professionals were assessed.

**Results:**

Among 14 survey participants, 4 correctly answered the relationships of 6 symptom pairs (28.57%). Two participants could not correctly conduct the calculations involved in mood disorder diagnoses (14.29%). Eleven (78.57%) correctly indicated that 2 or more sets of criteria were available for single diagnoses of mental illnesses. Only 1 (7.14%) correctly answered that the associations between symptoms and diagnoses were supported by including symptoms in the diagnostic criteria of the diagnoses. Nine (64.29%) correctly answered that the diagnosis variances were not fully explained by their symptoms. The confidence of participants in the *major depressive disorder* diagnosis and the willingness to take medications for this diagnosis were the same (mean = 5.50, standard deviation [*SD*] = 2.31). However, the confidence of participants in the symptom-based diagnosis of *non-solid brain tumor* was significantly lower (mean = 1.62, *SD* = 2.33, *p* < 0.001).

**Conclusion:**

Our study found that mental illness diagnoses are wrong from the perspectives of the public because our participants did not agree with all the assumptions professionals make about mental illness diagnoses. Only a minority of our participants obtained correct answers to the calculations involved in mental illness diagnoses. In the literature, neither patients nor the public have been engaged in formulating the diagnostic criteria of mental illnesses.

## Introduction

Mental illnesses are associated with a large global disease burden (1). In 2016, more than one billion people were affected by mental or addictive disorders (1). In terms of disability-adjusted life years, mental and addictive disorders account for 7% of the global disease burden in 2016 (1). To identify patients, mental illness diagnoses often are made based on symptoms (2). The Diagnostic and Statistical Manual of Mental Disorders (DSM) provides lists of symptoms that mental health professionals use to make diagnoses (3). However, these mental illness diagnoses are not without some concerns. For example, using the DSM or the International Classification of Diseases (ICD), different diagnosis criteria can coexist for the same diagnoses (4–6). Consistent principles regrading symptom selection and symptom duration are lacking with respect to formulating diagnostic criteria across diagnoses (7). Moreover, an overlap in symptoms across diagnoses is not uncommon (7). In addition, the role of trauma may be undervalued in diagnoses (7). Thus, some have argued that mental illness diagnoses are scientifically meaningless (8).

In addition, symptom-based diagnostic criteria are composite measures subject to problems that undermine their validity (9–11). The diagnoses of three common mental illnesses, dysthymic disorder, major depressive episodes (for the diagnosis of major depressive disorder or bipolar disorder according to the DSM, 4th edition, text revision [DSM-IV-TR]), and manic episodes (for the diagnosis of bipolar disorder), are, in fact, complicated mathematical equations that use data processing procedures that introduce biases into the diagnoses (9). Under most circumstances, the diagnoses of these three illnesses cannot be fully explained by their own input symptoms (9). In other words, biases have been introduced to these three diagnoses with few exceptions (9).

In addition, several implicit assumptions are embedded in mental illness diagnostic criteria. The prevalence of these three diagnoses are determined by the diagnostic criteria, input symptom prevalence, and symptom correlations (9). Although the major or minor criteria for mental illness diagnosis seem to suggest the relative importance of all symptoms, certain input symptoms in the minor criteria are unexpectedly more important than the others (9).

Recently, awareness has grown concerning patient perspectives about mental health, particularly mental health care and quality of care (12). Nevertheless, we are worried that the perspectives of patients and the public are still lacking with respect to mental illness diagnoses. The DSM 5 diagnoses have been criticized for a lack of recognizing individual experiences (7). Moreover, our study did not find any relevant studies that used the public’s perspective to assess the diagnostic criteria of mental illnesses. Thus, the present study aims to assess the DSM diagnostic criteria from the public’s perspective by using an anonymous survey.

## Materials and Methods

Beginning in 2021, our pilot study made an anonymous survey available online (take this fun survey below before continuing reading)^1^. We developed this survey based on recent studies concerning the assumptions made about mental illness diagnostic criteria (9). The survey had 30 questions in total. Ten questions focused on participants’ perceptions about, or confidence in mental illness diagnoses. The ratings ranged from 0 to 10. The other 20 questions related to the assumptions professionals make about mental illness diagnostic criteria (9). The survey questions about these assumptions, particularly the relationships between symptoms and diagnoses, were derived from the results in a publication (9). The survey questions about the equations that represent the diagnostic criteria of three mental illnesses were based on published information (9). The equations depict how information about symptoms is used to generate diagnoses (9). Based on these equations, participants were invited to do the calculations and obtain diagnoses using the presence and absence of input symptoms. The survey questions concerning the relationships between mental symptoms were based on the DSM-IV-TR criteria. Correct or suggested answers to the 20 questions were obtained from relevant literature.

We posted survey invitations to the public on social media. We provided the purpose of the survey and an introduction to the survey questions on the consent page. Survey participation was completely voluntary, and withdrawal was allowed at any time. We did not ask questions about participants’ demographic characteristics or personal information that could be used to identify individuals, including names, job titles, addresses, and Internet Protocol (IP) addresses. We asked one question about whether they were mental health professionals to assess whether they had in-depth knowledge about mental illness diagnostic criteria, but not to identify them as the individuals.

### Data Management and Analysis

We summarized continuous variables as mean values and standard deviations (SDs) and compared medians using the Wilcoxon rank sum test (13, 14). We summarized categorical variables in percentages. We considered a two-tailed p value less than 0.05 as statistically significant. We conducted data management and statistical analyses with R (v4.0.3) (15) and RStudio (v 1.4.1106) (16).

### Ethics Review

This study was reviewed and approved by the Veritas Independent Review Board (2021-2804-7063-7). We conducted our survey in accordance with the Declaration of Helsinki. Only adults were allowed to participate as specified in the informed consent form^2^. All participants provided consent for research use.

## Results

Among 14 survey participants, 11 answered all the questions (79%). None of the respondents were mental health professionals, and all knew that mental illness diagnoses often were made based on symptoms (100%).

### Perception of the Role of Symptoms

We assessed participants’ perceptions about the role of mental symptoms using several questions (see Table 1). First, we asked them whether the roles of six pairs of symptoms were the same or otherwise. All participants answered these six questions (100%), and four correctly answered all of them (29%).

**TABLE 1:** Participants’ perception about the assumed relationships between mental symptoms.

Symptom pairs	Correct or suggested answers	Incorrect answers
Q5: “sleep too much” and “insomnia”	(1) Same role	(2) Opposite
N	9	5
%	64.29%	35.71%
***Solution: “sleep too much” and “insomnia” are two symptoms that constitute a criterion in the minor criteria for the diagnosis of major depressive***		
***episodes and dysthymic disorder***		
Q6: “decreased need for sleep” and “insomnia”	(1) Different roles	(2) Same role
N	7	7
%	50%	50%
***Solution: “decreased need for sleep” is a symptom in the minor criteria for the diagnosis of manic episodes; “insomnia” is a symptom in the minor***		
***criteria for the diagnosis of major depressive episodes and dysthymic disorder***		
Q7: “depressed mood” and “diminished interest or pleasure”	(1) Different roles	(2) Same role
N	7	7
%	50%	50%
***Solution: “depressed mood” is a symptom in the major criteria and “diminished interest or pleasure” is a symptom in the minor criteria for the***		
***diagnosisof major depressive episodes***		
Q8: “unintentional weight loss” and “unintentional weight gain”	(1) Same role	(2) Different roles
N	9	5
%	64.29%	35.71%
***Solution: “unintentional weight loss” and “unintentional weight gain” are two symptoms that constitute a criterion in the minor criteria for the***		
***diagnosis of major depressive episodes***		
Q9: “poor appetite” and “overeating”	(1) Same role	(2) Different roles
N	9	5
%	64.29%	35.71%
***Solutions: “poor appetite” and “overeating” are two symptoms that constitute a criterion in the minor criteria for the diagnosis of dysthymic disorder***		
Q10: “poor concentration” and “distractibility”	(1) Different roles	(2) Same role
N	7	7
%	50%	50%
***Solution: “poor concentration” is a symptom in the minor criteria for the diagnosis of dysthymic disorder; “distractibility” is a symptom in the minor***		
***criteria for the diagnosis of manic episodes***		
Q5–Q10	All correct	Incorrect, at least once
N	4	10
%	28.57%	71.43%

*Diagnostic criteria of major depressive episodes, dysthymic disorder, and manic episodes based on the Diagnostic and Statistical Manual of Mental Disorders, 4th Edition, Text Revision (29).*

### Calculations Involved in the Diagnosis

We asked participants to do the calculations involved in the diagnosis of the three conditions. Two participants replied with incorrect answers for all the three calculations (14.29%, Table 2). Five participants (35.71%) considered these calculations closely related to the mental illness diagnoses. Two participants did not answer which equations represented mental illness diagnoses (14.29%). Five (35.71%) correctly indicated the three diagnoses represented by the equations.

**TABLE 2:** Calculations involved in the diagnosis of major depressive episodes, dysthymic disorder, and manic episodes.

Calculations for the diagnosis	Correct answers	Incorrect answers		
(Q11) Calculation for the diagnosis of major depressive episodes: 1 × 0 × (1 + 1 + 1 + 0 + 1 + 0- 3) + (1- 1 × 0) × (1 × 0) × (1 + 1 + 1 + 0 + 1 + 0 + 1–4)	(1) Answer is 0	(2) Answer is 1		
N	12	2		
%	85.71%	14.29%		
(Q12) Calculation for the diagnosis of dysthymic disorder: 1 × 0	(1) Answer is 0	(2) Answer is 1		
*N*	12	2		
%	85.71%	14.29%		
(Q13) Calculation for the diagnosis of manic episodes: (1- 1 × 0) × (1 + 0) × 1 × (1 + 0 + 0 + 0 + 0 + 1 + 1-3) + (1 – (1 – 1 × 0) x (1 + 0)) × 1 × (1 + 0 + 0 + 0 + 0 + 1 + 1–3)	(1) Answer is 0	(2) Answer is 1		
*N*	12	2		
%	85.71%	14.29%		
Q11– Q13	All correct	Incorrect, at least once		
*N*	12	2		
%	85.71%	14.29%		
(Q14) Calculations closely related to diagnoses	(1) Closely related	(2) Not related		
N	5	9		
%	35.71%	64.29%		
(Q15) The diagnosis represented by the equation: A_ma1 × A_ma2 × (A_mi3 + A_mi4 + A_mi5 + A_mi6 + A_mi7 + A_mi8 + A_mi9 + A_bias1) + (1- A_ma1 × A_ma2) × (me_ma1 × A_ma2) × (A_ mi3 + A_mi4 + A_mi5 + A_mi6 + A_mi7 + A_mi8 + A_mi9 + A_bias2)	(1) Major Depressive Episodes	(2) Dysthymic Disorder	(3_) Manic episodes	NA
*N*	6	5	1	2
%	42.86%	35.71%	7.14%	14.29%
(Q16) The diagnosis represented by the equation: A_ma × A_mi	(1) Dysthymic Disorder	(2) Major Depressive Episodes	(3) Manic Episodes	NA
*N*	7	4	1	2
%	50%	28.57%	7.14%	14.29%
(Q17) The diagnosis represented by the equation: (1- A_ma1 × A_ma2) × (A_ma1 + A_ma2) × A_ma3 × (A_mi1 + A_ mi2 + A_mi3 + A_mi4 + A_mi5 + A_mi6 + A_mi7 + A_bias1) + (1 – (1 – A_ma1 × A_ma2)(A_ma1 + A_ma2)) × A_ma3 × (A_mi1 + A_ mi2 + A_mi3 + A_mi4 + A_mi5 + A_mi6 + A_mi7 + A_bias2)	1. Manic Episodes	2. Dysthymic Disorder	3. Major Depressive Episodes	NA
*N*	5	3	4	2
%	35.71%	21.43%	28.57%	14.29%
Q15–Q17	All correct	Incorrect, at least once	NA	
*N*	5	7	2	
%	35.71%	50%	14.29%	

*NA, no answer.*

*Equations published elsewhere (9).*

### Assumptions About Mental Illness Diagnoses

We asked participants about the assumptions underlying mental illness diagnoses (Table 3). Eleven (78.57%) correctly indicated that two or more sets of criteria were available for single diagnoses of mental illnesses. Only 1 (7.14%) correctly answered that the association between symptoms and diagnoses was supported by making sure that the diagnostic criteria of the diagnosis included these symptoms. Four (28.57%) wrongly indicated that this causal relationship needed to be proved by examining the strengths of association between the diagnosis and the symptoms. Eight (57.14%) wrongly indicated that the causal inference should be made by looking for pathological or biological evidence. Nine (64.29%) correctly answered that the diagnosis variances could not be fully explained by its symptoms. Thirteen (92.86%) correctly indicated that mental symptoms are more common than diagnoses, assuming similar symptom prevalence and correlations. Only one participant (7.14%) correctly answered all four questions concerning assumptions about mental illness diagnoses.

**TABLE 3:** Assumptions about mental illness diagnoses.

Assumptions of mental illness diagnoses	Correct answers	Incorrect answers		
(Q18) Single set of diagnostic criteria for mental illnesses	(1) No, 2 or more sets of criteria for a single diagnosis.	(2) Of course, 1 set for an illness.		
*N*	11	3		
%	78.57%	21.43%		
***Solution: at least 3 sets of diagnostic criteria coexist: the Diagnostic and Statistical Manual of Mental Disorders (DSM), the International Statistical***				
***Classification of Diseases (ICD), and the Research Domain Criteria (RDoC, an approach by the National Institute of Mental Health)*** (26, 70).				
(Q19) Causation of symptoms by illnesses	(1) Make sure the diagnostic criteria of the diagnosis include these symptoms	(2) Exam the strengths of association between the diagnosis and these symptoms	(3) Look for pathological or biological evidence to understand the relationship between the diagnosis and the symptoms	NA
*N*	1	4	8	1
%	7.14%	28.57%	57.14%	7.14%
***Solution: symptoms are important measures to identify disorders and the evidence to support the causation between diagnoses and symptoms***				
***may be insufficient.*** (22, 55).				
(Q20) Diagnoses fully explained by symptoms	(1) NOT fully explained by symptoms	(2) Fully explained by symptoms	NA	
N	9	4	1	
%	64.29%	28.57%	7.14%	
***Solution: the diagnoses of major depressive episodes, dysthymic disorder, and manic episodes cannot be fully explained by their symptoms, assuming***				
***symptoms occurring with similar prevalence and similar correlations.***(9) ***One exception is the diagnosis of dysthymic disorder that can be fully explained***				
***by its symptoms when the symptoms are randomly assigned to 70% of the population*** (9).				
(Q21) Mental symptoms more common than diagnoses	(1) Yes.	(2) No.	NA	
N	13	0	1	
%	92.86%	0%	7.14%	
***Solution: the diagnoses of major depressive episodes, dysthymic disorder, and manic episodes occur less often than their input symptoms, assuming***				
***similar symptom prevalence and correlations.***(9)				
Q18–Q21	All correct	Incorrect, at least once		
N	1	13		
%	7.14%	92.86%		

*NA, no answer.*

### Symptoms Explaining Most of the Diagnosis Variances

**FIGURE 1:**
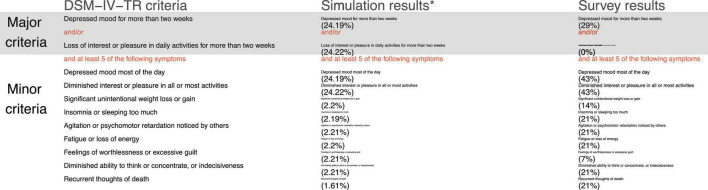
The symptoms that best explain the diagnosis of major depressive episodes based on R-squared and participants’ ratings. DSM-IV-TR, Diagnostic and Statistical Manual of Mental Disorders, 4th edition, text revision. Participants’ ratings, the proportions of all participants selecting the symptoms. *Percentages are the R-squared statistics representing the proportions of the variances of the diagnosis of major depressive episodes explained by the symptoms, assuming symptom prevalence as 0.3 and symptom correlations as 0.1 (9).

**FIGURE 2:**
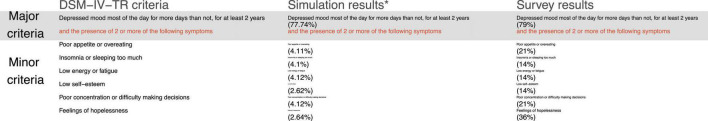
The symptoms that best explain the diagnosis of dysthymic disorder based on *R*-squared and participants’ ratings. DSM-IV-TR, Diagnostic and Statistical Manual of Mental Disorders, 4th edition, text revision. Participants’ ratings, the proportions of all participants selecting the symptoms. *Percentages are the *R*-squared statistics representing the proportions of the variances of the diagnosis of dysthymic disorder explained by the symptoms, assuming symptom prevalence as 0.3 and symptom correlations as 0.1 (9).

**FIGURE 3:**
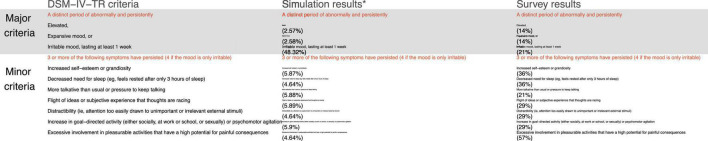
The symptoms that best explain the diagnosis of manic episodes based on *R*-squared and participants’ ratings. DSM-IV-TR, Diagnostic and Statistical Manual of Mental Disorders, 4th edition, text revision. Participants’ ratings, the proportions of all participants selecting the symptoms. *Percentages are the *R*-squared statistics representing the proportions of the variances of the diagnosis of manic episodes explained by the symptoms, assuming symptom prevalence as 0.3 and symptom correlations as 0.1 (9).

The symptoms that explained most of the variances of the diagnoses were assessed by using R-squared in a published study (9) and participant ratings in our survey. The R-squared for the symptoms explaining the diagnosis variances was obtained from simulations assuming symptom prevalence as 0.3 and symptom correlations as 0.1 (9). In Figures 1–3, the DSM-IV-TR criteria are listed and the text sizes of the symptoms are proportional to *R*-squared and participants’ ratings (proportions of participants selecting these symptoms). The *R*-squared and participants’ ratings for the symptoms explaining most of the variances of the diagnosis of major depressive episodes do not match in Figure 1. The symptom, “*loss of interest or pleasure in daily activities*,” in the major criteria was not considered as explaining most of the variances of the diagnosis by any participants (0%), but the *R*-squared was estimated to be 24.22%, which was higher than other symptoms. When we asked participants to choose whether “*depressed mood*” or “*loss of interest or pleasure*” explained more variances of the diagnoses of major depressive episodes, assuming a similar symptom prevalence, eight (57.14%) correctly answered “*it depends.”*

In Figure 2, the R-squared and participants’ ratings for the symptoms explaining most of the variances of the diagnosis of dysthymic disorder do not match, particularly for the symptoms in the minor criteria. In Table 4, when asked to choose the major or minor criteria that explained most of the variances of the diagnosis of dysthymic disorder, eight (57.14%) correctly chose the major criteria as explaining most of the variances of the diagnosis.

**TABLE 4:** Participants’ perception of the symptoms that better explain the diagnoses, assuming a similar symptom prevalence.

Symptoms better explaining diagnosis	Correct answers	Incorrect answers	
(Q23) “Depressed mood” or “loss of interest or pleasure” better explaining the diagnosis of major depressive episodes*	(1) It depends	(2) One of them	NA
*N*	8	4	2
%	57.14%	28.57%	14.29%
(Q25) The major or minor criteria better explaining the diagnosis of dysthymic disorder*	(1) Major criteria	(2) Minor criteria	NA
N	8	4	2
%	57.14%	28.57%	14.29%
(Q27) “Elevated,” “expansive,” or “irritable mood” better explaining the diagnosis of manic episodes*	(1) Only 1 of the 3	(2) Equally	NA
*N*	5	7	2
%	35.71%	50%	14.29%
Q23, Q25, and Q27	All correct	Incorrect, at least once	NA
*N*	2	10	2
%	14.29%	71.43%	14.29%

*Assuming the input symptoms occurring with similar prevalence and correlations (9).

In Figure 3, the *R*-squared and participants’ ratings for the symptoms explaining most of the variances of the diagnosis of manic episodes do not match, particularly for “*irritable mood*” in the major criteria and the symptoms in the minor criteria. In Table 4, when asked to choose which one of the three symptoms in the major criteria explained most of the variances of the diagnosis of manic episodes, five (35.71%) correctly chose only one of them as explaining more of the variances of the diagnosis, and seven (50%) incorrectly chose that these three symptoms equally explained the variances of the diagnosis.

Overall, only two participants (14.29%) correctly chose the symptoms that explained most of the variances of the three diagnoses.

### Confidence in the Diagnosis

Participants rated their confidence in the diagnoses and the willingness to take medications to treat the associated symptoms using a scale from 0, *not confident at all* or *not willing at all*, to 10, *very confident* or *very willing without conditions* (Table 5). We invited participants to rate their confidence in two symptom-based diagnoses, *major depressive disorder* and *non-solid brain tumor*. According to an estimate, the medications to control symptoms are assumed to be effective for 40–60% of the patients, while placebo worked for 20–40% of patients (17). Confidence in the *major depressive disorder* diagnosis and the willingness to take medications for this diagnosis were the same (mean = 5.50, *SD* = 2.31). However, the confidence in the diagnosis of “*non-solid brain tumor*” was significantly lower (mean = 1.62, *SD* = 2.33, *p* < 0.001), and the willingness to take medications for this diagnosis was not significantly different (mean = 3.38, *SD* = 3.52, *p* = 0.1) from that of “*major depressive disorder.*” Confidence in the diagnosis of mental illnesses in the end of the survey was not significantly lower (mean = 3.92, *SD* = 2.72, *p* = 0.16) than the confidence in the diagnosis of “*major depressive disorder.*”

**TABLE 5:** Confidence in symptom-based diagnoses and the willingness to take medications for symptom control.

Confidence on the diagnosis (0–10, from not confidence at all to very confident)	Statistics					
(Q3) Confidence on the diagnosis of major depressive disorder based on symptoms^#^	*N*	Mean	*SD*	Median	Min	Max
	14	5.50	2.31	5.5	1	9
(Q4) Willingness to take medication for symptoms*	*N*	Mean	*SD*	Median	Min	Max
	14	5.50	2.31	5.5	1	9
(Q28) Confidence on the diagnosis of non-solid brain tumor based on symptoms^#^	*N*	Mean	*SD*	Median	Min	Max
	13	1.62	2.33	1	0	7
(Q29) Willingness to take medication for symptoms*	*N*	Mean	*SD*	Median	Min	Max
	13	3.38	3.52	2	0	9
(Q30) Confidence on the diagnosis of mental illnesses in the end of the survey^∧^	*N*	Mean	*SD*	Median	Min	Max
	13	3.92	2.72	5	0	8

*^#^p < 0.001, based on the Wilcoxon rank sum test.*

*p = 0.1, based on the Wilcoxon rank sum test. The medications were assumed to have the same efficacy to treat symptoms of the diagnoses: 40–60% effectiveness for patients, while placebo worked for 20% to 40% of the patients (17).

*^∧^p = 0.16, compared with the confidence in the diagnosis of major depressive disorder based on symptoms (Q3) using the Wilcoxon rank sum test. SD, standard deviation.*

Overall, no participants correctly answered all the 20 questions related to the assumptions about mental illness diagnoses.

## Discussion

For more than a decade, mental illness diagnoses have been called wrong for several reasons, including the lack of validity (18, 19) and an insufficient evidence base (20–23). Some researchers have called for the abolition of the current diagnostic approach (24, 25). Some of the issues involved in the mental illness diagnoses, long discussed by psychiatrists, are the unclear boundaries between mental illnesses, overlaps between diagnostic categories (26, 27), and poor specificity (28–30). Moreover, the disorders that some diagnoses aim to identify may not exist at all (27). The present study has found that mental illness diagnoses are wrong from the public’s perspectives, since these diagnoses are built on various assumptions, many of which lack evidence to support and which do not align with the public’s perceptions about mental illnesses. None of our participants correctly identified all the assumptions underlying mental illness diagnoses, and only a minority were able to obtain the correct answers to the calculations involved in mental illness diagnoses.

### Symptom Reporting Assumptions

For symptom-based diagnoses, the basic assumptions are that symptoms are reported accurately, interpreted in the same manner by both patients and clinicians, and documented precisely for making diagnoses. These assumptions do not seem to hold well. For example, symptoms are not accurately reported by patients with anxiety (31) or arrhythmia (32). In retrospective settings, symptom reporting accuracy can be biased (33), including the pain and dyspnea reported in experimental settings (34).

Symptom interpretation by clinicians is important, since it can influence treatment choices (35). However, symptoms are not likely to be objective measures, since patients and clinicians do not necessarily interpret or understand them in the same way. In several studies that looked at the symptoms reported by both patients and clinicians, the agreement between patient-reported and clinician-reported symptoms was low, particularly with respect to cancer patients (36–40).

In the present study, the public did not interpret symptoms the same way as professionals. Less than 30% of the participants agreed with the professionally assumed relationships in six pairs of symptoms for the diagnosis of three mood disorders. One prominent example was that half of our survey participants considered “*poor concentration*” the same as “*distractibility*,” even though these two symptoms are used for exactly opposite diagnoses—dysthymic disorder and manic episodes—respectively.

Moreover, patient-reported symptoms may not be well documented by clinicians, even for well-defined symptoms, such as chest pain, dyspnea, and cough (41). This problem is exacerbated by the differences in symptom interpretation among clinicians (42, 43). Mental illness diagnoses are particularly problematic, since few biomarkers are available for most diagnoses to verify the accuracy of symptom reporting or documentation.

### Relationships Between Symptoms

The relationships between symptoms (i.e., statistical correlations) are an important assumptions that not only determines the prevalence of a diagnosis, but also have important effects on the overlap and correlations among diagnoses (9). Several factors can influence symptom correlations, for example, whether patients or clinicians consider them similar or interchangeable and whether they occur more often in certain patients. Overall, evidence is lacking regarding the correlations among symptoms that are used to diagnose mental illnesses (9). In our survey, participants did not consider that the six pairs of symptoms had the same relationships. Their attitudes toward the symptom pairs varied and subsequently influenced how often they reported these symptoms together.

More interestingly, the symptom pairs of different degrees of correlations have been used to construct single criteria items. For example, “*insomnia*” and “*sleeping too much*” are a pair of symptoms that do not occur together as often as “*fatigue*” and “*loss of energy*,” and these two pairs of symptoms are considered similarly important to the diagnosis of dysthymic disorder (3). Thus, some studies have critiqued this lack of consistency in symptom selection for different criteria (7, 28). However, the diagnostic criteria used to diagnose mood disorder are designed to have several symptoms in common that will lead to correlations between diagnoses. For example, a “*decreased need for sleep*” and “*insomnia*” are two symptoms that some participants considered the same, although clinicians use for opposite diagnoses, manic episodes and major depressive episodes, respectively; clinicians also use the two symptoms, “*distractibility*” and “*diminished ability to concentrate*” that some participants considered the same to make these two opposite diagnoses (3). Similar symptoms for major depressive episodes and manic episodes can lead to correlations between these two diagnoses (9), so we hypothesize that some patients may be diagnosed with bipolar disorder simply due to the design of the diagnostic criteria. In simulations, when the symptoms, “*distractibility*” and “*diminished ability to concentrate*” occurred randomly in small proportions of populations, the risk of co-occurrence of both major depressive episodes and manic episodes existed simply due to the design of diagnostic criteria (9).

One neglected assumption concerning the relationships among symptoms is to put more weight on the symptoms that constitute single items of the major or minor criteria than on the symptoms that form pairs. For example, the symptom pair, “*insomnia*” or “*sleeping too much*,” is regarded as important for a diagnosis of major depressive episodes and dysthymic disorder as single symptoms, such as “*recurrent thoughts of death.*” Thus, having symptoms of “*insomnia*” and “*sleeping too much*” at the same time has the same diagnostic value as having “*recurrent thoughts of death.*” In such circumstances, any two symptoms used to form items of the major or minor criteria are given less weights.

Lastly, little information is available on the rationale for the weighting schemes imposed on the items (symptom pairs or single symptoms) of the major or minor criteria. In the minor criteria for the diagnosis of major depressive episodes, dysthymic disorder, and manic episodes, different items are given the same weights. This is a strong assumption for outcome prediction. When symptoms are used to predict outcomes in regression models, their regression coefficients are likely to vary in different magnitudes. In contrast, when symptoms are summed together as a diagnosis for outcome prediction, the regression coefficients of the input symptoms can in fact be represented by the coefficient of the diagnosis (44, 45). This strategy assumes that the effect sizes of these items are the same for various outcomes (10). Imposing such assumptions on medical diagnoses is imposing restrictions on the relationships between symptoms, which can lead to indices or diagnoses that fail to predict major outcomes, particularly mortality, more accurately than their input symptoms or the biases generated by inadequate data processing (10, 11).

### Composite Diagnostic Criteria Are Equations

Until recently, mental illness diagnoses were not recognized as composite diagnostic criteria that work as complicated equations that integrate information from input symptoms (9). The evidence to support the design of composite criteria for mental illnesses seems to be lacking. For example, the formats of the equations representing three diagnoses—major depressive episodes, dysthymic disorder, and manic episodes—are assumed to be distinct. The structures of these three diagnostic criteria are assumed different, but the evidence to support this strategy is not clear (7).

Although the diagnostic criteria can be transformed precisely into equations (9), less than 40% of our survey participants correctly linked the equations with the diagnoses that these equations represent. When the absence and presence of the input symptoms currently used by professionals in the equations were replaced with 0 and 1 s, respectively, not all the participants could do the calculations correctly and choose the correct answers. Thus, we hypothesized that the differences in the capacity to solve complicated equations may be one of the reasons why mental illness diagnoses are not consistently made between professionals (42).

### Other Assumptions

It is widely accepted by clinicians that more than one set of diagnostic criteria can apply to single mental illnesses, particularly when using the DSM and the ICD systems (26). In fact, several versions of the DSM and ICD provide very different perspectives on how we define mental illnesses (26). In contrast, more than 20% of our participants believed there should be one set of diagnostic criteria for a single diagnosis.

Although the causes of mental illnesses have been well discussed (46), how and by what magnitudes mental illnesses cause their symptoms have not been well studied. A recent study indicated that not all mental symptoms are significantly correlated with the diagnoses of mood disorders that they aim to confirm (9). Currently, the real-world epidemiological evidence to support the associations between symptoms and diagnoses seems insufficient. In the present study, more than half of our participants considered obtaining pathological or biological evidence is the best approach to establish causal relationships between symptoms and diagnoses. Less than 8% approved using diagnostic criteria for causal inference.

In addition to causal relationships, symptom-based diagnoses may not be explained fully by their symptoms due to the complicated diagnostic criteria that often distort the relationships between symptoms and diagnoses (10, 45). However, in the present study more than 25% of our participants thought that diagnoses should be explained fully by their symptoms.

One related assumption of the diagnostic criteria is the implicit limitations on diagnosis prevalence. For example, in the minor criteria for the diagnosis of three mood disorders, the requirement of having multiple symptoms at the same time can lead to diagnoses less prevalent than their input symptoms (9).

Composite diagnostic criteria implicitly assume that patients with the same diagnoses are subject to similar treatment, since a common underlying cause has been identified (11). For example, the exercise and nutritive interventions have been applied to frail patients, regardless of their symptoms used to fulfill the diagnostic criteria (11). Exercise and nutritive interventions have been used to treat patients without physical and nutritive deficits, respectively (or both) (11). For major depressive disorder, hypersomnia and insomnia are assumed to be caused by the same underlying condition (3). This suggests that this disorder is likely to have interventions that treat both hypersomnia and insomnia, since the underlying cause is the same. However, the choices of medications for sleep disturbance in patients with major depressive disorder partly depend on the presence of hypersomnia or insomnia (47). Patients’ responses to anti-depressants have been found to be related to the sleep symptoms they present and can be attributed to different pathological mechanisms (47), but the diagnosis of major depressive disorder remains similar from DSM-III to DSM-5. The diagnostic criteria for mental illnesses, at least major depressive disorder, seem insensitive to patients’ responses to currently available medications and underlying pathological mechanisms.

### Perception of Symptoms

The symptoms of the DSM diagnostic criteria are presented in an order that may suggest their importance. For example, the symptoms are grouped in the major and minor criteria for three mood disorders (9). Those in the major criteria may be regarded as more important than those in the minor criteria. However, patients or the public may not perceive symptoms’ importance in the order they are presented in the diagnostic criteria. The present study found that the symptoms of the major criteria for diagnosing manic episodes are considered less important for explaining the diagnosis than those of the minor criteria. For example, our participants considered one of the symptoms of the major criteria for diagnosing major depressive episodes— “*loss of interest or pleasure*” —unimportant to the diagnosis.

The design of diagnostic criteria puts more weights on certain symptoms that are not necessarily those in the major criteria or those that our survey participants considered more important (9). Assuming similar symptom prevalence and correlations, patterns of the importance of the symptoms for explaining the diagnoses have been observed (9). When choosing from 2 or 3 symptoms, less than 20% of our participants were able to correctly select the symptoms that explained more of the variances of the three diagnoses in our survey. How patients rate the importance of various mental symptoms and report them accordingly have not been well studied. With respect to a patient with acute myocardial infarction (48, 49) or ovarian cancer (50), their interpretation of their symptoms can influence their health care-seeking behaviors. Whether patients’ perspectives influence mental symptom reporting and thus the diagnostic accuracy needs to be studied in the future.

### Confidence in the Diagnosis

Few studies are available on individuals’ confidence in the diagnoses of mental illnesses, compared to the many studies on attitudes toward mental illness and care-seeking behavior (51). The present study found that confidence in the mental illness diagnoses and medications seems to be influenced by individuals’ understanding of how mental illnesses are diagnosed and what these diagnoses are called. Mental illness diagnoses have been framed as biomedical labels (52). Many researchers think mental illnesses have a biological basis (53), such as mood disorders, Alzheimer’s disease, and Down syndrome (27). The Research Domain Criteria also assume that mental illnesses are brain disorders (54). However, few mental illness diagnoses are actually called diseases that represent distinct processes of human biology (55). The present study found that the confidence in symptom-based diagnoses depends on whether they are called a “*major depressive disorder*” or a “*non-solid brain tumor.*” Although some psychiatrists consider that the DSM provides a biomedical framing of mental illnesses (52), our participants were significantly less confident in the name of a symptom-based diagnosis that suggested biological roots. In contrast, the willingness to take medications for symptom control did not significantly vary based on what the diagnoses were called. Participants seemed less concerned with the diagnosis or the label of their conditions and said they would take medications for symptom control. Moreover, after going through the questions and reviewing the diagnostic criteria for mental illnesses, participants’ confidence in the mental illness diagnoses did not decrease significantly.

### Public and Patient Disengagement

Patient and public engagement has become an essential part of the evaluation of health technologies, since patients’ perspectives provide information that may help to improve the technologies under evaluation, and some of the patient-reported outcomes are not less important than those assessed by clinicians (56). The levels of patient engagement is directly linked to patients’ health care experiences (57) and is associated with health care practice and treatment decisions in primary care (58). Essentially, ethical imperatives exist to hear patients’ perspectives on emerging health technologies (56). Some professionals have suggested that the development of current DSM approaches is not firmly based on patients’ perspectives (59). The findings of the present study suggest a lack of public or patient involvement in formulating the diagnostic criteria for mental illnesses. Important assumptions that members of the public are likely to disagree with have not been actively exposed to, or discussed by, health professionals during the DSM revision process. Some professionals have begun to consider patients’ input as important to the diagnosis of mental illnesses (12).

Moreover, the American Psychiatric Association (APA), the publisher of the DSM, has been proud of its explicit exclusion of non-health care professionals from participating in the DSM-5 Working Groups that formulate the diagnostic criteria for mental illnesses (60). Although the Working Groups invited external advisors to participate and more than 100 conferences were held (60), the public’s perspectives were considered only regarding a few select issues (61, 62). The public comments that the DSM-5 sought needed to be based on the relevant literature and secondary data analysis by professionals or researchers (63), which did not reflect the public’s or patients’ opinions and attitudes toward diagnostic criteria. The assumptions that the DSM-5 is built upon have not been exposed to patients’ and the public’s scrutiny. Thus, the legitimacy of the DSM has been put in doubt due to the lack of patient participation in the formation of its diagnostic criteria (64). This lack of patient engagement results in diagnostic criteria that are filled with assumptions and presumed relationships between symptoms with which the public may disagree.

### Composite Diagnostic Criteria Are Problematic

Composite diagnostic criteria that aggregate information from multiple symptoms or signs have been used widely in various medical diagnoses, including frailty (65) and mental illnesses (9, 10, 44, 45). Until recently, composite diagnostic criteria have not been considered problematic (11). Then, data scientists began assessing composite diagnostic criteria by rewriting the diagnostic criteria of mental illnesses into equations (9). From a mathematical perspectives, these equations can be complicated, and the calculations may not always be done correctly, particularly the diagnosis of manic episodes (see Table 2) (9). The methods to aggregate information from various input symptoms often induce biases by censoring sums of input variables or categorizing continuous variables (10). These biases partly explain why distinct populations may be considered the same and receive similar treatment (11). Recent evidence also indicate that the use of composite diagnostic criteria of poor interpretability is associated with early terminations of clinical trials (66).

These problems are more controllable if the composite diagnostic criteria are executed precisely and used with sufficient reliability amongst clinicians. However, the APA officially encourages clinicians to examine patients’ social, psychological, and biological factors and to use these factors for case formulation (67). In other words, diagnoses should consider implicit factors not mentioned in the DSM criteria. In reality, the reliability of case formulation varies across settings and awaits improvement (68). The reliability of case formulation does not seem good enough (69). In addition to the biases embedded in the DSM criteria (9), case formulation adds another layer of information that cannot be explained by symptoms alone.

In conclusion, if symptom-based diagnostic criteria are valid, reliable, and accurate enough, why are they not used to diagnose all medical conditions? Recent evidence shows that the problematic assumptions of the diagnostic criteria of mental illnesses may have been overlooked by mental health professionals (9). Based on the responses to our survey questions, the public’s perspectives and perceptions to symptoms do not align with the assumptions of the diagnostic criteria held by professionals. Thus, current diagnostic approach has various shortcomings that threaten its validity. However, when professionals’ careers and large sums of money are at stake (59), the incentives to change the system of mental illness diagnoses remains weak. Facing poor incentives for an overhaul, we think our results important to foster fundamental changes in the diagnostic criteria of mental illnesses.

### Limitations

The conclusion that mental illness diagnoses are right or wrong is a judgment or an opinion, rather than a testable hypothesis. In the present study, we considered how the public interprets the diagnoses of mental illnesses and their confidence in symptom-based diagnoses using an online survey that involved technical terms in mental illnesses. This perspective is very different from that of mental health professionals who design, frame, and use mental illness diagnoses. Before the implementation of our study, we aimed to include professionals in our survey and have a question they could answer to self-identify whether they were mental health professionals. However, the recruitment was challenging and we lacked the resources to incentivize professionals to participate. Some mental health care professionals may think the public’s perspective fails to prove mental illness diagnoses wrong. We agree that this critique has its own basis and is an opinion based on mental health care professionals’ perspectives. This pilot study is a first attempt, with a limited sample size, to show professionals that their current diagnostic approach may be regarded wrong by the public. We will continue examining mental illness diagnoses using professionals’, patients’, and the public’s perspectives.

Moreover, the diagnostic criteria have been shifting from DSM-IV-TR to DSM-5 (29). Although the diagnosis of manic episodes has been modified in the DSM-5 (54), many of the shortcomings of the diagnosis remain relevant, including the arbitrary and implicit weights put on the symptoms of the major criteria.

## Conclusion

The diagnostic criteria of mental illnesses are based on various assumptions, many of which lack the evidence to support them, and which do not match the expectations of the public. For example, the assumed relationships between symptoms in six symptom pairs were not agreed by all our participants. Symptoms for the diagnosis of opposite mood disorders could be considered the same by public members. Symptom pairs of different degrees of correlations have been used to construct single items for diagnoses. Symptoms used to construct items for diagnosis have been implicitly given less weights than the symptoms used as single items. In the recent literature, diagnoses of mental illnesses have been recognized as composite diagnostic criteria that are complicated equations that integrate information from input symptoms.

In our study, a minority of our participants correctly linked the equations to the diagnoses they represented. Moreover, not all participants could correctly do the equation calculations. Not all participants agreed that there could be more than one set of diagnostic criteria for a single mental illness. Less than 8% approved using diagnostic criteria for causal inference. More than 25% thought diagnoses should be explained fully by their symptoms, although simulations proved otherwise. The symptoms used to diagnose mental illnesses are ordered based on their assumed importance. However, our participants considered some symptoms in the major criteria as not important at all for the diagnosis of mood disorders.

In our survey, confidence in the mental illness diagnoses and medications seems to be influenced by our participant’ understanding of how mental illnesses are diagnosed and whether the diagnosis is suggestive of biological roots. Participants were significantly less confident in a symptom-based diagnosis called “*non-solid brain tumor*,” compared with “*major depressive disorder.*”

The formulation of diagnostic criteria for mental illnesses lacks patient and public engagement. Recent evidence shows that the composite diagnostic criteria that the DSM uses to design mental illness diagnoses introduce biases into the diagnoses, link distinct populations to the same diagnosis, and may be associated with early terminations of trials. It is unclear when the DSM will begin to accept patients’ and the public’s perspectives, and understand the biases embedded in its composite diagnostic criteria.

## Data Availability Statement

The raw data supporting the conclusions of this article will be made available by the authors, without undue reservation.

## Ethics Statement

The studies involving human participants were reviewed and approved by Veritas Independent Review Board (2021-2804-7063-7). Written informed consent for participation was not required for this study in accordance with the national legislation and the institutional requirements.

## Author Contributions

Y-SC conceptualized and designed this study, managed, and analyzed data and drafted the manuscript. C-JW assisted in data management and computation. Y-CL, H-TH, Y-PC, H-CW, S-YH, and W-CC participated in the design of this study. All the authors reviewed and approved the manuscript.

## Conflict of Interest

Y-SC is employed by the Canadian Agency for Drugs and Technologies in Health. Y-SC conducted this study as an independent researcher out of academic curiosity without any material support. The remaining authors declare that the research was conducted in the absence of any commercial or financial relationships that could be construed as a potential conflict of interest.

## Publisher’s Note

All claims expressed in this article are solely those of the authors and do not necessarily represent those of their affiliated organizations, or those of the publisher, the editors and the reviewers. Any product that may be evaluated in this article, or claim that may be made by its manufacturer, is not guaranteed or endorsed by the publisher.

## Footnotes

1 https://docs.google.com/forms/d/e/1FAIpQLSdlulKwH5EigpuA9DKXc_KNrVPAnlErj9ioFMuqm5yZH7qTWQ/viewform?usp=sf_link or https://forms.gle/pT8W6zAY9kxgd8k59

2
https://docs.google.com/forms/d/e/1FAIpQLSdlulKwH5EigpuA9DKXc_KNrVPAnlErj9ioFMuqm5yZH7qTWQ/viewform?usp=sf_link
